# Changes in Heart Rate Associated with Exenatide Once Weekly: Pooled Analysis of Clinical Data in Patients with Type 2 Diabetes

**DOI:** 10.1007/s13300-018-0370-z

**Published:** 2018-02-03

**Authors:** Steven P. Marso, Elise Hardy, Jenny Han, Hui Wang, Robert J. Chilton

**Affiliations:** 1 Research Medical Center, Kansas City, MO USA; 2 AstraZeneca, Gaithersburg, MD USA; 3 Bristol-Myers Squibb/AstraZeneca, San Diego, CA USA; 4 University of Texas Health Science Center, San Antonio, TX USA

**Keywords:** Exenatide once weekly, Glucagon-like peptide-1 receptor agonists, Heart rate, Tolerability, Type 2 diabetes

## Abstract

**Introduction:**

Glucagon-like peptide-1 receptor agonists (GLP-1RAs) improve glycemia in patients with type 2 diabetes, but heart rate increases have been observed.

**Methods:**

A pooled post hoc analysis of 11 randomized clinical trials (*N* = 4595) of 10–30 weeks’ duration from the exenatide once-weekly (QW) development program evaluated heart rate with exenatide QW (intervention group) and exenatide twice daily (BID), liraglutide, and non-GLP-1RAs (insulin, metformin, pioglitazone, and sitagliptin) (comparison groups). The time course and size of heart rate changes from baseline and the relationship of heart rate change with baseline heart rate were studied. A multivariate analysis (9 studies; *N* = 3903) examined associations between patient characteristics or treatments and heart rate increases.

**Results:**

Mean baseline heart rate ± standard deviation was 75.0 ± 8.5 beats per minute (bpm) with exenatide QW (*n* = 2096), 75.8 ± 8.7 bpm with exenatide BID (*n* = 606), 75.2 ± 8.9 bpm with liraglutide (*n* = 450), and 74.5 ± 8.6 bpm with non-GLP-1RAs (*n* = 1443). Least-squares mean ± standard error changes from baseline to final heart rate were + 2.7 ± 0.2, + 1.0 ± 0.3, and + 3.0 ± 0.4 bpm with exenatide QW, exenatide BID, and liraglutide, respectively, and − 0.8 ± 0.2 bpm with non-GLP-1RAs. The size and direction of heart rate changes in individual patients varied within each treatment group at all time points. At posttreatment follow-up, heart rate reverted to the baseline level after GLP-1RA discontinuation. Heart rate changes correlated negatively with baseline heart rate for all therapies (*r* = − 0.3 to − 0.4). Baseline heart rate was the strongest predictor of increased heart rate.

**Conclusions:**

Small increases in heart rate were associated with exenatide QW, exenatide BID, and liraglutide treatments but reverted to baseline after discontinuation. Increases were more likely in patients with a low baseline heart rate. The clinical relevance of these heart rate increases is unknown but will be clarified by several ongoing and recently completed cardiovascular outcome studies.

**Electronic supplementary material:**

The online version of this article (10.1007/s13300-018-0370-z) contains supplementary material, which is available to authorized users.

## Introduction

Glucagon-like peptide-1 receptor agonists (GLP-1RAs) are indicated with diet and exercise for the improvement of glycemic control in adults with type 2 diabetes mellitus (T2DM), many of whom are taking background oral glucose-lowering medications such as metformin and sulfonylureas. Exenatide was the first-in-class GLP-1RA and is available worldwide in twice-daily (BID) and once-weekly (QW) formulations for administration via subcutaneous injection. The slow pharmacokinetics and inherent titration of the extended-release formulation achieves minimal effective concentrations in 2 weeks and steady-state concentrations in 6–7 weeks, compared with the intermittent exposure of exenatide achieved with BID dosing. Liraglutide is a GLP-1RA that is available as a once-daily (QD) subcutaneous injection, and maximum concentrations are achieved 8–12 h postdose [1].

GLP-1RAs reduce glucose levels by multiple mechanisms, including glucose-dependent stimulation of insulin secretion, suppression of glucagon, and slowing of gastric emptying [2]. In randomized controlled studies, exenatide BID and QW have also been associated with improvements in cardiovascular risk factors, including body weight, blood pressure, lipids, and endothelial function [3–8]. In addition, exenatide has been shown to not prolong the QTc interval at supratherapeutic concentrations [9, 10]. Liraglutide QD has also been associated with improved body weight, systolic blood pressure, and triglycerides [6, 11, 12] and a reduced risk of cardiovascular events [13].

The use of GLP-1RAs is associated with increases in heart rate [14]. The aim of this post hoc analysis was to examine the relationship of heart rate changes over time, to explore the relationship of heart rate changes with baseline heart rate, and to identify patient characteristics associated with changes in heart rate in the exenatide QW development program.

## Methods

### Study Selection and Pooling

Data from 11 randomized controlled clinical studies in the exenatide QW development program conducted between 2005 and 2011 were included in this analysis (Electronic Supplementary Material [ESM] Table S1) [3, 4, 8, 15–22]. Studies were of 10–30 weeks’ duration and compared exenatide QW with an active comparator (exenatide BID, liraglutide, insulin, metformin, pioglitazone, or sitagliptin; 9 studies) or placebo (2 studies) in patients with T2DM. Data were analyzed for exenatide QW, exenatide BID, and liraglutide, pooled GLP-1RAs, pooled non-GLP-1RAs (insulin, metformin, pioglitazone, and sitagliptin), and individual non-GLP-1RA treatments. Patients receiving placebo (*n* = 23) were not pooled with active comparators.

### Data Collection

Vital sign data across all studies were collected using standard clinical trial procedures (sitting heart rate after 5 min of rest before other procedures). Heart rate (beats per minute [bpm]) was measured at baseline (see “Key Definitions”) and during all subsequent visits. In some studies with a ≥ 26-week treatment period, heart rate was also measured 10 weeks after the last treatment (posttreatment, “follow-up”) (ESM Table S1).

### Key Definitions

“Baseline” heart rate was defined as the average of all heart rate measurements for each patient before receiving the first randomized study medication. The “final” heart rate was defined as the last heart rate measurement for each patient while on treatment. The “posttreatment” heart rate was defined as the heart rate measurement taken at 10 weeks’ posttreatment in the studies with a treatment period of ≥ 26 weeks in which this was assessed. For the multivariate regression analysis, the “average postbaseline” heart rate was defined as the average of all postbaseline heart rate measurements in each patient.

“Antihypertensive medications” were defined based on the anatomic therapeutic chemical classifications of the World Health Organization drug dictionary (volume 26, 2012; http://www.who.int/medicines/publications/druginformation/en/), which included β-blockers, calcium channel blockers, agents acting on the renin–angiotensin system, diuretics, and other antihypertensive agents.

### Statistical Analysis

Demographics and baseline characteristics were summarized descriptively for the intention-to-treat populations. Mean heart rate and standard error were calculated at each time point. Heart rate at the end of treatment was imputed with the last observation carried forward method to include data for early withdrawals. Exenatide QW, exenatide BID, liraglutide, sitagliptin, pioglitazone, insulin, metformin, and placebo were all evaluated for heart rate changes. Data for exenatide QW, exenatide BID, and liraglutide were pooled for the GLP-1RA subgroup analysis, while data for sitagliptin, pioglitazone, insulin, and metformin were pooled for the non-GLP-1RA subgroup analysis.

To account for differences in individual baseline heart rate, we applied analysis of covariance models to evaluate the difference in the change in heart rate from baseline to the end of treatment between the exenatide QW, exenatide BID, liraglutide, and each individual non-GLP-1RA treatment group, and between the pooled GLP-1RA and pooled non-GLP-1RA treatments. The estimated least-squares (LS) mean heart rate changes were computed for treatment groups of interest, and the *P* values were derived. The mean heart rate change after the 10-week off-treatment follow-up period was assessed descriptively. All reported *P* values are nominal as there was no adjustment for multiplicity.

A multivariate analysis was conducted to identify patient characteristics associated with increases in heart rate from baseline. The data for this analysis came from nine studies, each of 24–30 weeks’ duration. The mean heart rate change postbaseline, calculated as the difference between the baseline heart rate and the average postbaseline heart rate at weeks 10, 14, 18, 22, 26, or 30 when data were available, was examined in a stepwise regression, weighted by the number of observations used to compute the average postbaseline heart rate in each patient. Treatment effect was examined for exenatide QW, exenatide BID, and liraglutide using the pooled non-GLP-1RA treatments (insulin, metformin, pioglitazone, and sitagliptin) as the reference group. Variables for demographics, baseline characteristics, prior and baseline glucose-lowering therapy (metformin and sulfonylurea combination vs. others), antihypertensive medication use at baseline (yes vs. no), and postbaseline changes in blood pressure and body weight were examined. Closely correlated variables were excluded from modeling to avoid collinearity. The base model included treatment group, baseline heart rate, and study identifier. All other covariates were then subject to a stepwise model selection, and the model with the smallest Akaike information criterion value was selected as the best model.

Statistical analyses were performed with R (version 3.1.2; http://www.R-project.org/) for the multivariate analysis and SAS (version 9.2; SAS Institute, Inc., Cary, NC, USA) for all other analyses.

### Compliance with Ethics Guidelines

This article is based on previously conducted studies and does not involve any new studies of human or animal subjects performed by any of the authors.

## Results

### Patient Populations

Baseline characteristics were generally similar among patients treated with exenatide QW (*n* = 2096), exenatide BID (*n* = 606), liraglutide (*n* = 450), pooled GLP-1RAs (*n* = 3152), or non-GLP-1RAs (*n* = 1443) in the overall population (Table 1), but the mean duration of T2DM was less among patients receiving non-GLP-1RAs. Most patients in all active treatment groups were male, with a mean body mass index of 30–32 kg/m^2^ and mean glycated hemoglobin of approximately 8.5%. Mean baseline heart rates were broadly similar for patients treated with GLP-1RAs (75.0 ± 8.5, 75.8 ± 8.7, and 75.2 ± 8.9 bpm with exenatide QW, exenatide BID, and liraglutide, respectively; 75.2 ± 8.6 bpm with pooled GLP-1RAs) and non-GLP-1RAs (74.5 ± 8.6 bpm). Demographic and baseline characteristic data for all individual treatment groups are shown in ESM Table S2.

**Table 1:** Demographic and baseline characteristics for the individual GLP-1RA groups and the pooled GLP-1RA and non-GLP-1RA groups

Patient characteristic	Treatment groups				
	Exenatide QW (*n* = 2096)	Exenatide BID (*n* = 606)	Liraglutide (*n* = 450)	GLP-1RAs (*n* = 3152)	Non-GLP-1RAs (*n* = 1443)
Age (years)	55.4 ± 10.3	55.5 ± 10.0	55.9 ± 9.6	55.5 ± 10.2	54.5 ± 10.7
Male	1192 (56.9)	326 (53.8)	245 (54.4)	1763 (55.9)	853 (59.1)
Race					
White	1042 (49.7)	173 (28.5)	287 (63.8)	1502 (47.7)	789 (54.7)
Asian	747 (35.6)	344 (56.8)	56 (12.4)	1147 (36.4)	429 (29.7)
Other	255 (12.2)	61 (10.1)	104 (23.1)	420 (13.3)	173 (12.0)
Black	52 (2.5)	28 (4.6)	3 (0.7)	83 (2.6)	52 (3.6)
Weight (kg)	86.3 ± 20.7	82.8 ± 21.4	91.1 ± 19.1	86.3 ± 20.8	86.0 ± 19.2
Body mass index (kg/m^2^)	30.9 ± 5.8	30.0 ± 5.7	32.3 ± 5.4	30.9 ± 5.7	31.0 ± 5.5
HbA1c (%)	8.5 ± 1.1	8.5 ± 1.1	8.4 ± 1.0	8.5 ± 1.1	8.5 ± 1.1
Duration of T2DM (years)	7.1 ± 5.6	7.8 ± 5.6	8.8 ± 6.5	7.4 ± 5.8	5.4 ± 5.4
Heart rate (bpm)	75.0 ± 8.5	75.8 ± 8.7	75.2 ± 8.9	75.2 ± 8.6	74.5 ± 8.6
Systolic BP (mmHg)	130.9 ± 14.6	129.9 ± 14.2	133.0 ± 13.3	131.0 ± 14.3	130.8 ± 15.5
Diastolic BP (mmHg)	78.9 ± 9.1	78.9 ± 9.0	79.6 ± 8.6	79.0 ± 9.0	79.6 ± 9.1
Baseline antihypertensive medications					
Agents acting on RA system	800 (38.2)	123 (20.3)	273 (60.7)	1196 (37.9)	486 (33.7)
β-Blockers	278 (13.3)	39 (6.4)	115 (25.6)	432 (13.7)	173 (12.0)
Calcium channel blockers	333 (15.9)	80 (13.2)	84 (18.7)	497 (15.8)	191 (13.2)
Diuretics	232 (11.1)	27 (4.5)	70 (15.6)	329 (10.4)	172 (11.9)
Other antihypertensive agents	60 (2.9)	8 (1.3)	12 (2.7)	80 (2.5)	49 (3.4)
Background glucose-lowering therapy^a^					
Diet and exercise	314 (15.0)	46 (7.6)	7 (1.6)	367 (11.6)	547 (37.9)
Metformin	833 (39.7)	159 (26.2)	129 (28.7)	1121 (35.6)	664 (46.0)
Metformin + SU	700 (33.4)	273 (45.0)	271 (60.2)	1244 (39.5)	141 (9.8)
Metformin + SU + TZD	16 (0.8)	12 (2.0)	1 (0.2)	29 (0.9)	1 (< 0.1)
Metformin + TZD	124 (5.9)	32 (5.3)	21 (4.7)	177 (5.6)	69 (4.8)
SU	61 (2.9)	48 (7.9)	17 (3.8)	126 (4.0)	1 (< 0.1)
SU + TZD	20 (1.0)	17 (2.8)	1 (0.2)	38 (1.2)	0 (0.0)
TZD	10 (0.5)	11 (1.8)	0 (0.0)	21 (0.7)	1 (< 0.1)

Data are presented as a number with the percentage in parenthesis or as the mean ± standard deviation

*BID* twice daily, *BP* blood pressure, *bpm* beats per minute, *GLP*-*1RA* glucagon-like peptide-1 receptor agonist, *HbA1c* glycated hemoglobin, *QW* once weekly, *RA* renin–angiotensin, *SU* sulfonylurea, *T2DM* type 2 diabetes mellitus, *TZD* thiazolidinedione

^a^Background therapies used by < 1% of patients in all groups are not shown

The posttreatment follow-up analysis included data from three studies, each with a treatment period of ≥ 26 weeks (ESM Table S1) [8, 16, 22]. At baseline, there were 2254 patients in this subgroup, with 971, 261, and 450 of these patients receiving exenatide QW, exenatide BID, and liraglutide, respectively, 1682 in the pooled GLP-1RA group, and 572 patients receiving a non-GLP-1RA active treatment. Posttreatment follow-up data were available for a total of 2062 patients who had received exenatide QW (*n* = 901), exenatide BID (*n* = 243), liraglutide (*n* = 411), or a non-GLP-1RA treatment (*n* = 507); the pooled GLP-1RA group for posttreatment follow-up included 1555 patients.

The multivariate regression analysis included data from nine studies, each with a treatment period of 24–30 weeks (ESM Table S1). Patients for whom a full set of observations for dependent and independent variables was available were assessed in the regression models, which included 3903 patients.

### Changes in Mean Heart Rate

**Fig. 1:**
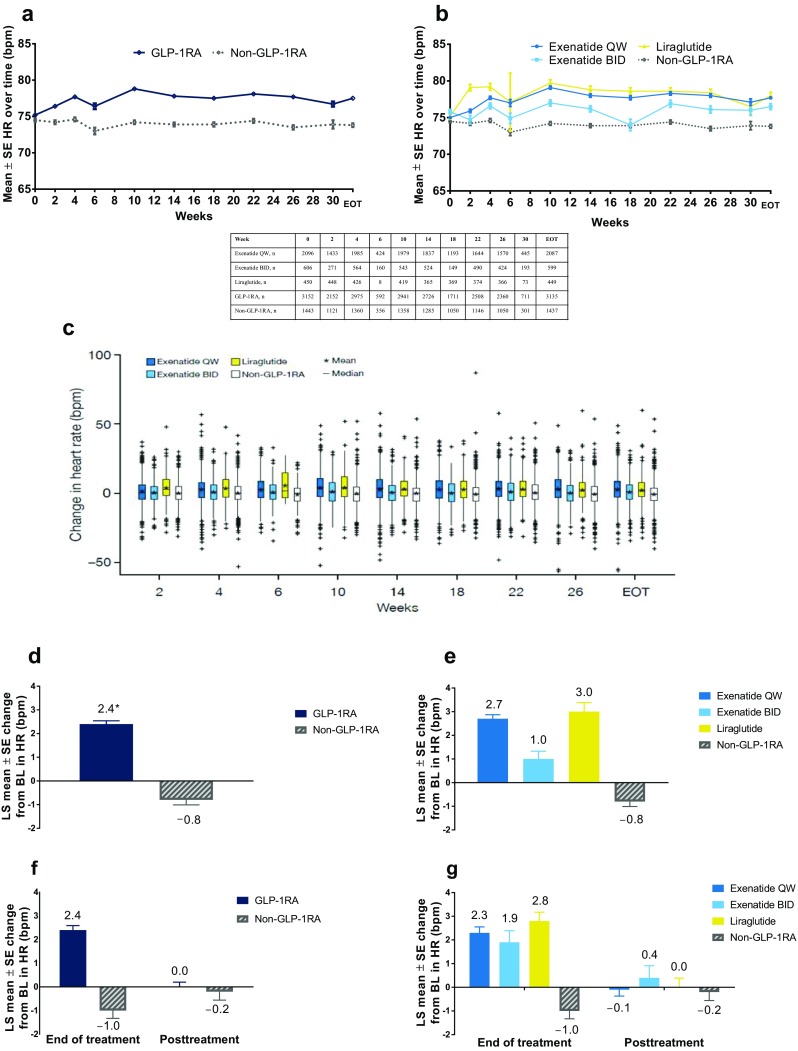
** a**,** b** Mean (± SE) HR over time: analysis by GLP-1RAs vs. non-GLP-1RA treatments (**a**) and by individual GLP-1RAs vs. non-GLP-1RA treatments (**b**). **c** Box and whisker plot of change in HR from baseline at each time point.** d**,** e** LS mean (± SE) change in HR from baseline to final on-treatment HR measurement by GLP-1RAs vs. non-GLP-1RA treatments (**d**) and by individual GLP-1RAs vs. non-GLP-1RA treatments (**e**). **f**, **g** LS mean (± SE) change in HR over time in studies with posttreatment follow-up for GLP-1RA vs. non-GLP-1RA treatments (**f**) and individual GLP-1RAs vs. non-GLP-1RA treatments (**g**). *P < 0.0001 vs. non-GLP-1RA treatments. *BID* twice daily,* BL* baseline *bpm* beats per minute, *EOT* end of treatment, *GLP*-*1RA* glucagon-like peptide-1 receptor agonist, *HR* heart rate, *LS* least-squares, *QW* once weekly, *SE* standard error

Data pooled from the controlled periods of all 11 studies showed fluctuations in mean heart rate over time across all treatments. Early increases in mean heart rate were observed by week 2 in the pooled GLP-1RA (mean change + 1.8 bpm), exenatide QW (+ 1.4 bpm), exenatide BID (+ 0.6 bpm), and liraglutide (+ 4.0 bpm) groups, while the mean heart rate remained stable over time in the pooled non-GLP-1RAs group (mean change at week 2: 0.0 bpm) (Fig. 1a, b). The size and direction of changes in heart rate from baseline varied within each treatment group at all time points, with some patients having decreases in heart rate while others in the same treatment group had increases (Fig. 1c).

After adjusting for individual patient’s baseline heart rate, the LS mean change in heart rate from baseline to end of treatment was greater with pooled GLP-1RAs (+ 2.4 bpm) than with non-GLP-1RAs (− 0.8 bpm) (Fig. 1d) and with exenatide QW (+ 2.7 bpm), exenatide BID (+ 1.0 bpm), and liraglutide (+ 3.0 bpm) (Fig. 1e). Data by individual treatment group are presented in ESM Fig. S1.

In the three studies with posttreatment follow-up periods, the trend in heart rate increases was similar between groups, but mean heart rate changes returned to near baseline values at 10 weeks posttreatment (Fig. 1f, g). Among patients treated with exenatide QW, exenatide BID, liraglutide, pooled GLP-1RAs, and pooled non-GLP-1RAs in these three studies, LS mean changes in heart rate from baseline to end of treatment were + 2.3, + 1.9, + 2.8, + 2.4, and − 1.0 bpm, respectively, and were − 0.1, + 0.4, 0.0, 0.0, and − 0.2 bpm, respectively, at the end of the 10-week posttreatment follow-up period.

### Relationship of Change in Heart Rate with Baseline Heart Rate

**Fig. 2:**
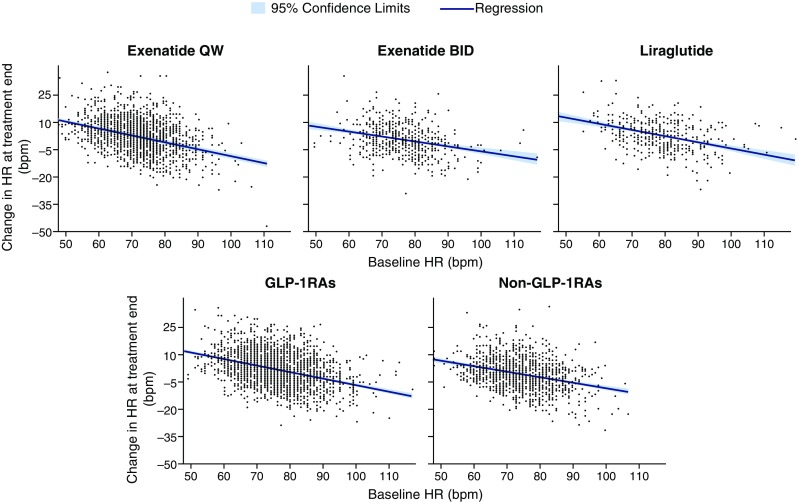
Regression for change in HR at treatment end vs. baseline HR

Change in heart rate at the end of treatment was negatively correlated with baseline heart rate for the exenatide QW (*r* = − 0.3680; *P* < 0.0001), exenatide BID (*r* = − 0.2898; *P* < 0.0001), liraglutide (*r* = − 0.3883; *P* < 0.0001), pooled GLP-1RAs (*r* = − 0.3572; *P* < 0.0001), and pooled non-GLP-1RAs (*r* = − 0.3132; *P* < 0.0001) treatment groups (Fig. 2). Data by individual treatment group are presented in ESM Fig. S2 and were consistent with the pooled data for non-GLP-1RAs.

### Multivariate Analysis

The selected best model included study identifier, treatment group, sex, Asian race, age, baseline heart rate, use of both sulfonylurea and metformin at baseline, use of antihypertensive medications at baseline, and weight change from baseline to study end point. The *R*^2^ of the selected model was 0.1899, meaning that 19% of the total variance in heart rate change was explained by the model. The variance explained by each individual factor ranged from 0.01% for weight change to 13.10% for baseline heart rate (Table 2).

**Table 2:** Estimated effect of patient characteristics and GLP-1RA treatment on change in heart rate

Covariate	Estimate (95% CI)	*P* value	Total variance explained (%)
Patient characteristics			
Male sex	0.3312 (− 0.13 to 0.79)	0.1581	0.17
Asian race	1.7447 (0.96–2.53)	0.0000	0.40
Baseline age (per year)	− 0.0413 (− 0.06 to − 0.02)	0.0005	0.03
Baseline heart rate (per beat)	− 0.3563 (− 0.38 to − 0.33)	0.0000	13.10
Baseline metformin + sulfonylurea use	1.4286 (0.82–2.04)	0.0000	0.46
Baseline β-blocker use	0.7373 (0.01–1.46)	0.0463	0.05
Weight change from baseline to end point (per kg)	0.0460 (− 0.01 to 0.11)	0.1377	0.01
Study identifier	–	–	1.74
Glucose-lowering treatment (vs. non-GLP-1RA therapy)			3.03
Exenatide BID	1.5761 (0.47–2.68)	0.0051	
Exenatide QW	3.7382 (3.07–4.41)	0.0000	
Liraglutide	4.0145 (2.81–5.22)	0.0000	
Residuals	–	–	81.01

*CI* confidence interval

### Change in Heart Rate in Patients with and without Prior β-blocker Treatment

**Fig. 3:**
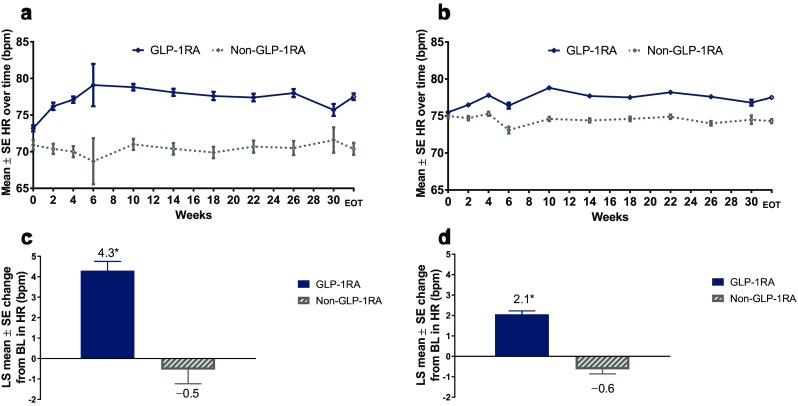
**a**,** b** Mean (± SE) HR over time with GLP-1RAs vs. non-GLP-1RA treatments in patients with prior β-blocker exposure (**a**) and β-blocker-naïve patients (**b**).** c**,** d** LS mean (± SE) HR change from baseline to final on-treatment HR measurement with GLP-1RAs vs. non-GLP-1RA treatments in patients with prior β-blocker exposure (**c**) and β-blocker-naïve patients (**d**). *P < 0.001 vs. non-GLP-1RA treatments

As observed in the full patient population, early increases in mean heart rate were observed by week 2 in the pooled GLP-1RA subgroup in both patients who were taking β-blockers at baseline and those who were β-blocker-naïve, while mean heart rate remained stable over time in the pooled non-GLP-1RAs group (Fig. 3a, b). Furthermore, in both β-blocker subgroups, the LS mean change in heart rate from baseline to end of treatment was greater with pooled GLP-1RAs than with pooled non-GLP-1RAs (Fig. 3c, d).

## Discussion

This analysis showed that, although heart rate measures were highly variable, small, reversible heart rate increases were rapidly observed among patients treated with exenatide QW, exenatide BID, and liraglutide compared with non-GLP-1RA therapies. Changes in heart rate varied markedly between patients within each treatment group. Not all patients experienced increases in heart rate, and some patients in each treatment group had no change or a reduction in heart rate from baseline at each time point. Heart rate changes were negatively correlated with baseline heart rate, regardless of treatment, with observed increases being greater in patients with a low heart rate at baseline and smaller in patients with a high heart rate at baseline, suggesting the phenomenon of regression to the mean. The mean heart rate over time remained within a normal range (< 80 bpm) among patients treated with GLP-1RAs.

Because β-blockers are known to reduce heart rate [23], changes in heart rate with GLP-1RA therapy were examined in patient subgroups according to baseline β-blocker use. Increases in heart rate were seen among patients who were treated with GLP-1RAs in both those taking β-blockers at baseline and β-blocker-naïve patients, whereas the heart rate remained stable in those receiving non-GLP-1RA treatments in both β-blocker subgroups. Increases in heart rate among GLP-1RA-treated patients were greater for those taking β-blockers at baseline than for β-blocker-naïve patients; however, these results are confounded by differences in patient characteristics. Baseline characteristics were not balanced across the β-blocker subgroups and because the β-blocker population was determined based on baseline β-blocker use, treatment effects on heart rate could potentially be confounded by new concomitant β-blocker use or changes in dosage during the study treatment period.

The mechanism by which GLP-1RAs increase heart rate among patients with diabetes has yet to be established [24]. However, proposed mechanisms include vagal depression, sympathetic activation [25, 26], increased levels of endogenous insulin [24, 27], and stimulation of glucagon-like peptide-1 receptors in myocytes in the sinoatrial node [28]. Recent studies in healthy volunteers and patients with T2DM have suggested that GLP-1RAs may increase the heart rate through a direct stimulatory effect on the sinoatrial node [25, 29], and the differential effects of GLP-1RAs on heart rate appear to be the result of differences in molecular structure as well as duration of action [25].

Increased heart rate appears to be dependent on the GLP-1RA dose, with an associated heart rate increase of 2–3 bpm observed in a meta-analysis of randomized controlled trials of exenatide and liraglutide [14].

Doses of liraglutide QD and exenatide QW are active throughout the day or longer, and in our analysis they were associated with greater mean heart rate increases than exenatide BID. The onset of heart rate changes appeared to be more rapid with liraglutide than with exenatide QW.

Reviews and analyses on the effect of GLP-1RAs on heart rate have also found differences between long- and short-acting formulations. One meta-analysis found that exenatide QW and liraglutide, but not exenatide BID, were associated with increases in heart rate in comparison to placebo [24]. A review that investigated the effects of GLP-1RAs on 24-h heart rate and stratified these GLP-1RAs by formulation found that long-acting formulations could induce a 24-h time-averaged mean heart rate increase of 3–10 bpm, while the short-acting formulations resulted in a transient (10–12-h) increase in heart rate of 8–10 bpm [25]. These results are consistent with an earlier analysis showing that short-acting GLP-1RAs (exenatide BID and lixisenatide QD) were associated with smaller, more transient increases in heart rate than the longer-acting GLP-1RAs exenatide QW, liraglutide, and albiglutide; while the increase in heart rate with dulaglutide was small, it persisted at nighttime [30]. These differences in heart rate increases between individual GLP-1RAs may be due to pharmacokinetic differences between short- and long-acting formulations [31].

The cardiovascular safety of T2DM therapies is of central importance because patients with T2DM are at increased risk of cardiovascular disease. The risk of cardiovascular events has been shown to increase with heart rate in epidemiological studies, although higher heart rate was not usually associated with drug therapy in these populations and the data were not stratified by baseline heart rate. Furthermore, patients with higher heart rate may have underlying health conditions, giving rise to potential selection bias in these epidemiological studies. An increase in the resting heart rate of 15 bpm has been found to be associated with an increased risk of cardiovascular disease mortality [11]. Increased heart rate could accelerate the progression to arterial wall stiffness and atherosclerosis [32]. However, the clinical importance of increased heart rate varies with a patient’s clinical circumstances.

Ongoing and recently completed cardiovascular safety outcome trials will help resolve questions surrounding the clinical implications of heart rate increases with GLP-1RA therapy. To date, published results from cardiovascular outcome trials have demonstrated that GLP-1RA treatment was either noninferior or superior to comparators in reducing cardiovascular end-point events in different patient populations with T2DM. The available data thus far do not indicate that GLP-1RA treatment, despite the associated increases in heart rate, is associated with an increased risk of cardiovascular events. The Liraglutide Effect and Action in Diabetes: Evaluation of Cardiovascular Outcome Results (LEADER) trial (NCT01179048), an international placebo-controlled study, evaluated the effect of liraglutide compared with placebo, added on to standard care, on cardiovascular events in 9340 patients with T2DM and high cardiovascular risk [13]. After a median follow-up of 3.8 years, liraglutide-treated patients had a significant reduction in the primary composite outcome of the first occurrence of cardiovascular death, nonfatal myocardial infarction (MI), or nonfatal stroke (13.0 vs. 14.9% for placebo; *P* < 0.01 for superiority). The Trial to Evaluate Cardiovascular and Other Long-term Outcomes With Semaglutide in Subjects With Type 2 Diabetes (SUSTAIN-6; NCT01720446) evaluated semaglutide or placebo, given in addition to standard care, in 3297 patients with T2DM and high cardiovascular risk [33]. After 104 weeks, semaglutide demonstrated noninferiority to placebo for the primary composite end point of the first occurrence of cardiovascular death, nonfatal MI, or nonfatal stroke (6.6 vs. 8.9%; *P* < 0.001 for noninferiority; *P* = 0.02 for superiority). The incidence of nonfatal stroke (1.6 vs. 2.7%; *P* = 0.04), but not cardiovascular death or nonfatal MI, was significantly lower with semaglutide than with placebo. Lixisenatide was compared with placebo among patients with T2DM who recently experienced an acute coronary event in the phase 3b Evaluation of Lixisenatide in Acute Coronary Syndrome (ELIXA) trial (NCT01147250) [34]. Lixisenatide demonstrated noninferiority to placebo for the primary outcome, time to the first occurrence of major adverse cardiovascular events, defined as cardiovascular death, nonfatal MI, nonfatal stroke, or hospitalization for unstable angina (hazard ratio [HR] 1.02; 95% confidence interval [CI] 0.89–1.17). No increased risk of heart failure (HR 0.96; 95% CI 0.75–1.23) or death (HR 0.94; 95% CI 0.78–1.13) was observed with lixisenatide. There was a mean increase in heart rate of + 0.4 bpm with lixisenatide versus placebo (*P* = 0.01).

Results from the Exenatide Study of Cardiovascular Event Lowering (EXSCEL; NCT01144338), a placebo-controlled phase 4 trial examining exenatide QW for effects on cardiovascular outcomes in 14,752 patients with T2DM, of whom more than 70% had prior cardiovascular disease, showed no increase in cardiovascular events compared to placebo [35]. Assessment of the primary outcome of this study, the time to the first confirmed cardiovascular event in the primary composite cardiovascular end point (cardiovascular death, nonfatal MI, or nonfatal stroke), over a median follow-up of 3.2 years indicated that exenatide QW was noninferior to placebo for safety (*P* < 0.001) but not superior to placebo for efficacy (*P* = 0.06) [35]. An LS mean increase in heart rate of + 2.51 bpm was observed with exenatide QW versus placebo during the study period (*P* < 0.001).

A meta-analysis of the LEADER, SUSTAIN-6, ELIXA, and EXSCEL trials found that GLP-1RAs significantly reduced the risk of the composite end point of cardiovascular death, nonfatal MI, or nonfatal stroke (*P* = 0.033), in addition to reducing cardiovascular mortality (*P* < 0.007) and all-cause mortality (*P* < 0.002) [36]. The effect of GLP-1RA therapy on cardiovascular outcomes will also be assessed in the Researching Cardiovascular Events With a Weekly Incretin in Diabetes (REWIND) trial (NCT01394952), which will compare the time to the first occurrence of cardiovascular death, nonfatal MI, or nonfatal stroke with dulaglutide versus placebo over an average follow-up of approximately 6.5 years. Results from this study are expected in 2018.

The limitations of the current analysis include its post hoc nature and the relatively short duration of the included studies. Limitations of the data are that heart rate measurements were part of clinical procedures for collecting vital signs for clinical trials and were therefore not a standardized procedure, as would occur in a clinical study focused on heart rate data. The collection of heart rate data also varied among studies because of different study schedules, resulting in variations in patient numbers for each time point. Because heart rate was measured during clinic visits, it was not possible to examine changes outside these visits or the general variability in heart rate within individual patients. Another limitation is the considerably larger number of patients included in the exenatide QW group compared with the other treatment groups after pooling studies and treatments. This imbalance may limit the interpretation of results, although data were reported for the individual GLP-1RAs to avoid the potential impact of this imbalance on results for the pooled GLP-1RAs. Limitations associated with the multivariate analysis include lack of adjustment for multiplicity and the use of postbaseline factors, such as change in body weight. Nevertheless, this was justified by the post hoc nature of the multivariate analysis and the difficulty of distinguishing the direct effect of study medication on heart rate change and the indirect effect of study medication on body weight change and subsequently heart rate change. Although only the potential effect of background treatment with metformin plus a sulfonylurea was investigated in the analysis, this was the most frequently used background therapy combination. Patients receiving background therapy with metformin plus a sulfonylurea were likely to have been older and to have had a longer duration and greater severity of diabetes than patients on monotherapy background treatments. Because of these limitations, the multivariate analysis to identify potential risk factors for increased heart rate should be considered hypothesis-generating and to require validation.

## Conclusions

This analysis showed that treatment with GLP-1RAs was associated with an increase in heart rate among patients with T2DM, although the heart rate remained within normal ranges. Baseline heart rate was the strongest predictor of heart rate changes; however, further studies are required to confirm the results from this multivariate analysis. While the clinical relevance of increases in heart rate with GLP-1RA therapy is unclear, the results from GLP-1RA cardiovascular outcome trials to date do not indicate that increased heart rate is associated with an increased rate of cardiovascular events.

## Electronic supplementary material

Below is the link to the electronic supplementary material.

Supplementary material 1 (PDF 352 kb)
