# Supplementary material for: Changes in Heart Rate Associated with Exenatide Once Weekly: Pooled Analysis of Clinical Data in Patients with Type 2 Diabetes
Source: Diabetes Ther. 2018 Feb 3;9(2):551–64. doi: 10.1007/s13300-018-0370-z (PMC6104267; doi:10.1007/s13300-018-0370-z)

SUPPLEMENTARY MATERIAL

Table S1 Summary of studies

Study	ITT, *N*	Randomized duration, weeks	Posttreatment follow-up,[a] Y/N	Treatment	Background diabetes treatment	Study visits, weeks
Kim et al.[b]	45	15	N	Exenatide QW 0.8 or 2.0 mg (*n* = 16, 15)<br>Placebo (*n* = 14)	MET	1, 2, 3, 4, 5, 6, 7, 8, 9, 10, 11, 12, 13, 14, 15
Drucker et al. (DURATION-1)	295	30	N	Exenatide QW 2.0 mg (*n* = 148)<br>Exenatide BID 5 μg (4 wk)→ 10 μg (*n* = 147)	MET/SU/TZD or any combination of 2 of these agents	1, 2, 3, 4, 6, 10, 14, 18, 22, 26, 30
Davies et al.	216	26	N	Exenatide QW 2.0 mg (*n* = 111)<br>Insulin detemir QD or BID (*n* = 105)	MET/MET+SU	4, 8, 12, 18, 26
Iwamoto et al.[b]	29	10	N	Exenatide QW 0.8 mg (*n* = 10)<br>Exenatide QW 2.0 mg (*n* = 9)<br>Placebo (*n* = 10)	BG/SU/TZD/BG+SU/ BG+TZD/SU+TZD	1, 2, 3, 4, 5, 6, 7, 8, 9, 10
Bergenstal et al. (DURATION-2)	491	26	N	Exenatide QW 2.0 mg (*n* = 160)<br>SITA 100 mg/day (*n* = 166)<br>PIO 45 mg/day (*n* = 165)	MET	1, 2, 4, 6, 10, 14, 18, 22, 26
Diamant et al. (DURATION-3)	456	26	N	Exenatide QW 2.0 mg (*n* = 233)	MET/MET+SU	1, 2, 4, 8, 14, 18, 22, 26

Study	ITT, *N*	Randomized duration, weeks	Posttreatment follow-up,[a] Y/N	Treatment	Background diabetes treatment	Study visits, weeks
				Insulin glargine 10 IU/d adjusted to achieve target glucose of 4.0–5.5 mmol/L (*n* = 223)		
Inagaki et al.	427	26	N	Exenatide QW 2.0 mg (*n* = 215)<br>Insulin glargine 4 U adjusted to achieve FBG <5.5 mmol/L (*n* = 212)	BG alone/BG+TZD/ BG+SU/BG+TZD+SU	1, 2, 4, 8, 12, 20, 26
Blevins et al. (DURATION-5)	252	24	N	Exenatide QW 2.0 mg (*n* = 129)<br>Exenatide BID 5 μg (4 wk)→10 μg (*n* = 123)	Drug-naïve/MET/SU/TZD or any combination of these agents	1, 4, 8, 14, 20, 24
Russell-Jones et al. (DURATION-4)	820	26	Y[a]	Exenatide QW 2.0 mg (*n* = 248)<br>MET 2000 mg/day (*n* = 246)<br>PIO 45 mg/day (*n* = 163)<br>SITA 100 mg/day (*n* = 163)	Drug-naïve	1, 2, 4, 8, 12, 16, 20, 26, 36
Buse et al. (DURATION-6)	911	26	Y[a]	Exenatide QW 2.0 mg (*n* = 461)<br>Liraglutide QD 1.8 mg (*n* = 450)	MET/SU/MET+SU/MET+PIO	1, 2, 4, 8, 14, 18, 22, 26, 36
Ji et al.	678	26	Y[a,c]	Exenatide QW 2.0 mg (*n* = 340)<br>Exenatide BID 5 μg (4 wk)→	MET/SU/TZD/MET+SU/ MET+TZD/SU+TZD	

**Study**	**ITT, *N***	**Randomized duration, weeks**	**Posttreatment follow-up,[a] Y/N**	**Treatment**	**Background diabetes treatment**	**Study visits, weeks**
				10 µg (*n* = 338)		

BID twice daily, *BG* biguanide, *FBG* fasting blood glucose, *ITT* intention-to-treat, *MET* metformin, *PIO* pioglitazone, *SITA* sitagliptin, *SU* sulfonylurea, *TZD* thiazolidinedione, *QD* once daily, *QW* once weekly, *wk* week

[a] Included 10-week off-treatment follow-up period

[b] Not included in multivariate analysis

[c] Excluded Japanese sites where patients continued through the extension treatment period

Table S2 Demographic and baseline characteristics by individual treatment group in the overall population

	Exenatide QW (*n* = 2096)	Exenatide BID (*n* = 606)	Liraglutide (*n* = 450)	Sitagliptin (*n* = 329)	Pioglitazone (*n* = 328)	Insulin (*n* = 540)	Metformin (*n* = 246)	Placebo (*n* = 23)
Age, years	55.4 ± 10.3	55.5 ± 10.0	55.9 ± 9.6	52.0 ± 10.8	53.9 ± 10.5	57.1 ± 10.0	53.1 ± 11.1	57.2 ± 9.0
Male	1192 (56.9)	326 (53.8)	245 (54.4)	180 (54.7)	176 (53.7)	343 (63.5)	154 (62.6)	9 (39.1)
Race								
White	1042 (49.7)	173 (28.5)	287 (63.8)	163 (49.5)	175 (53.4)	291 (53.9)	160 (65.0)	9 (39.1)
Asian	747 (35.6)	344 (56.8)	56 (12.4)	75 (22.8)	74 (22.6)	229 (42.4)	51 (20.7)	10 (43.5)
Other	255 (12.2)	61 (10.1)	104 (23.1)	68 (20.7)	62 (18.9)	19 (3.5)	24 (9.8)	3 (13.0)
Black	52 (2.5)	28 (4.6)	3 (0.7)	23 (7.0)	17 (5.2)	1 (0.2)	11 (4.5)	1 (4.3)
Weight, kg	86.3 ± 20.7	82.8 ± 21.4	91.1 ± 19.1	87.8 ± 19.5	87.0 ± 19.2	84.3 ± 18.9	85.9 ± 19.6	86.9 ± 24.3
Body mass index, kg/m^2	30.9 ± 5.8	30.0 ± 5.7	32.3 ± 5.4	31.8 ± 5.3	31.8 ± 5.5	30.2 ± 5.5	30.7 ± 5.5	31.8 ± 7.0
HbA1c, %	8.5 ± 1.1	8.5 ± 1.1	8.4 ± 1.0	8.5 ± 1.2	8.5 ± 1.2	8.4 ± 0.9	8.6 ± 1.2	8.1 ± 1.3
Duration of T2DM, years	7.1 ± 5.6	7.8 ± 5.6	8.8 ± 6.5	4.0 ± 4.3	4.3 ± 4.9	8.2 ± 5.8	2.7 ± 3.6	5.3 ± 4.4
Heart rate, bpm	75.0 ± 8.5	75.8 ± 8.7	75.2 ± 8.9	73.9 ± 7.4	74.6 ± 7.9	75.0 ± 9.5	73.9 ± 8.6	69.8 ± 6.2
Systolic BP, mm Hg	130.9 ± 14.6	129.9 ± 14.2	133.0 ± 13.3	128.1 ± 13.9	128.9 ± 14.6	134.6 ± 16.6	128.8 ± 15.1	130.1 ± 12.0
Diastolic BP, mm Hg	78.9 ± 9.1	78.9 ± 9.0	79.6 ± 8.6	79.4 ± 8.5	80.3 ± 8.5	79.7 ± 9.9	79.1 ± 9.1	77.7 ± 6.7
Baseline antihypertensive medications								
Agents acting on RA system	800 (38.2)	123 (20.3)	273 (60.7)	58 (17.6)	53 (16.2)	280 (51.9)	95 (38.6)	11 (47.8)
β-blockers	278 (13.3)	39 (6.4)	115 (25.6)	29 (8.8)	29 (8.8)	79 (14.6)	36 (14.6)	1 (4.3)
Calcium channel blockers	333 (15.9)	80 (13.2)	84 (18.7)	18 (5.5)	15 (4.6)	127 (23.5)	31 (12.6)	4 (17.4)
Diuretics	232 (11.1)	27 (4.5)	70 (15.6)	22 (6.7)	19 (5.8)	87 (16.1)	44 (17.9)	0 (0.0)
Other antihypertensive agents	60 (2.9)	8 (1.3)	12 (2.7)	6 (1.8)	4 (1.2)	32 (5.9)	7 (2.8)	0 (0.0)
Background glucose-lowering								

	Exenatide QW (*n* = 2096)	Exenatide BID (*n* = 606)	Liraglutide (*n* = 450)	Sitagliptin (*n* = 329)	Pioglitazone (*n* = 328)	Insulin (*n* = 540)	Metformin (*n* = 246)	Placebo (*n* = 23)
therapy								
Diet and exercise	314 (15.0)	46 (7.6)	7 (1.6)	154 (46.8)	159 (48.5)	1 (0.2)	233 (94.7)	6 (26.1)
Insulin	1 (<0.1)	0 (0.0)	0 (0.0)	0 (0.0)	1 (0.3)	0 (0.0)	3 (1.2)	0 (0.0)
MET	833 (39.7)	159 (26.2)	129 (28.7)	169 (51.4)	161 (49.1)	326 (60.4)	8 (3.3)	10 (43.5)
MET + insulin	2 (<0.1)	4 (0.7)	0 (0.0)	2 (0.6)	4 (1.2)	1 (0.2)	0 (0.0)	0 (0.0)
MET + other	3 (0.1)	1 (0.2)	1 (0.2)	1 (0.3)	0 (0.0)	0 (0.0)	2 (0.8)	0 (0.0)
MET + SU	700 (33.4)	273 (45.0)	271 (60.2)	0 (0.0)	1 (0.3)	140 (25.9)	0 (0.0)	2 (8.7)
MET + SU + insulin	4 (0.2)	1 (0.2)	2 (0.4)	0 (0.0)	0 (0.0)	1 (0.2)	0 (0.0)	0 (0.0)
MET + SU + other	4 (0.2)	2 (0.3)	0 (0.0)	0 (0.0)	0 (0.0)	0 (0.0)	0 (0.0)	0 (0.0)
MET + SU + TZD	16 (0.8)	12 (2.0)	1 (0.2)	1 (0.3)	0 (0.0)	0 (0.0)	0 (0.0)	0 (0.0)
MET + TZD	124 (5.9)	32 (5.3)	21 (4.7)	0 (0.0)	0 (0.0)	69 (12.8)	0 (0.0)	0 (0.0)
Other	0 (0.0)	0 (0.0)	0 (0.0)	2 (0.6)	0 (0.0)	1 (0.2)	0 (0.0)	0 (0.0)
SU	61 (2.9)	48 (7.9)	17 (3.8)	0 (0.0)	1 (0.3)	0 (0.0)	0 (0.0)	3 (13.0)
SU + other	2 (<0.1)	0 (0.0)	0 (0.0)	0 (0.0)	0 (0.0)	0 (0.0)	0 (0.0)	0 (0.0)
SU + TZD	20 (1.0)	17 (2.8)	1 (0.2)	0 (0.0)	0 (0.0)	0 (0.0)	0 (0.0)	1 (4.3)
SU + TZD + insulin	1 (<0.1)	0 (0.0)	0 (0.0)	0 (0.0)	0 (0.0)	0 (0.0)	0 (0.0)	0 (0.0)
TZD	10 (0.5)	11 (1.8)	0 (0.0)	0 (0.0)	1 (0.3)	0 (0.0)	0 (0.0)	1 (4.3)
TZD + other	1 (<0.1)	0 (0.0)	0 (0.0)	0 (0.0)	0 (0.0)	1 (0.2)	0 (0.0)	0 (0.0)

Data are presented as *n* (%) or mean ± standard deviation

BID twice daily, *BP* blood pressure, *bpm* beats per minute, *HbA1c* glycated hemoglobin, *MET* metformin, *QW* once weekly, *RA* renin–angiotensin, *SU* sulfonylurea, *T2DM* type 2 diabetes mellitus, *TZD* thiazolidinedione

Fig. S1 (a) Mean ± SE HR over time and (**b**) LS mean ± SE HR change from BL to final on-treatment measurement by treatment. *BID* twice daily, *BL* baseline, *bpm* beats per minute, *EOT* end of treatment, *HR* heart rate, *LS* least-squares, *QW* once weekly, *SE* standard error

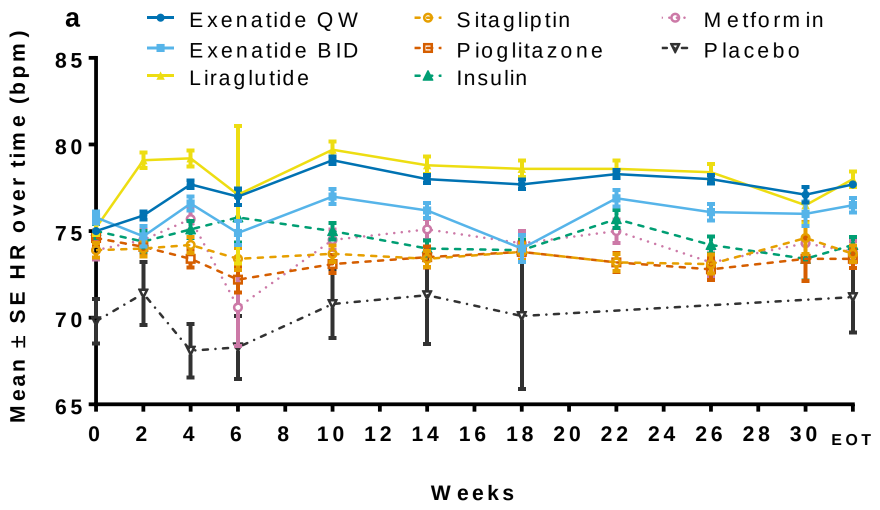

Week	0	2	4	6	10	14	18	22	26	30	EOT
Exenatide QW, n	2096	1433	1985	424	1979	1837	1193	1644	1570	445	2087
Exenatide BID, n	606	271	564	160	543	524	149	490	424	193	599
Liraglutide, n	450	448	426	8	419	365	369	374	366	73	449
Sitagliptin, n	329	325	315	160	310	296	286	275	227	77	325
Pioglitazone, n	328	327	308	158	298	283	268	258	226	52	328
Insulin, n	540	223	504	29	518	483	286	401	443	95	538
Metformin, n	246	246	233	9	232	223	210	212	154	77	246
Placebo, n	23	23	23	23	23	12	7	0	0	0	23

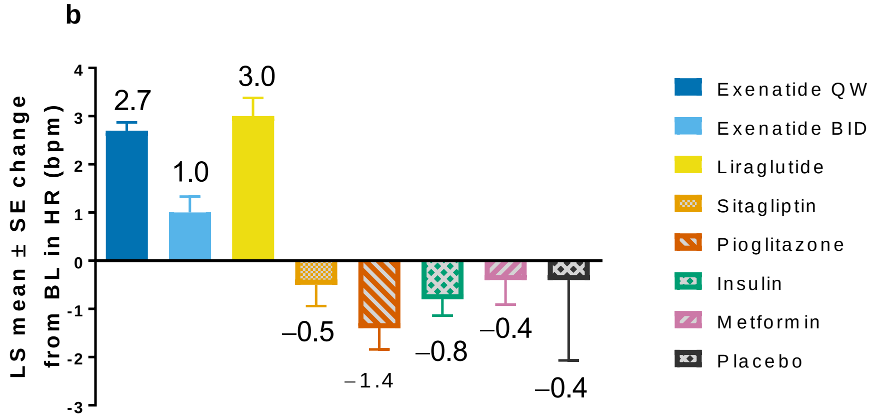

Fig. S2 Regression for change in HR at treatment end versus baseline HR by treatment. *BID* twice daily, *bpm* beats per minute, *HR* heart rate, *QW* once weekly

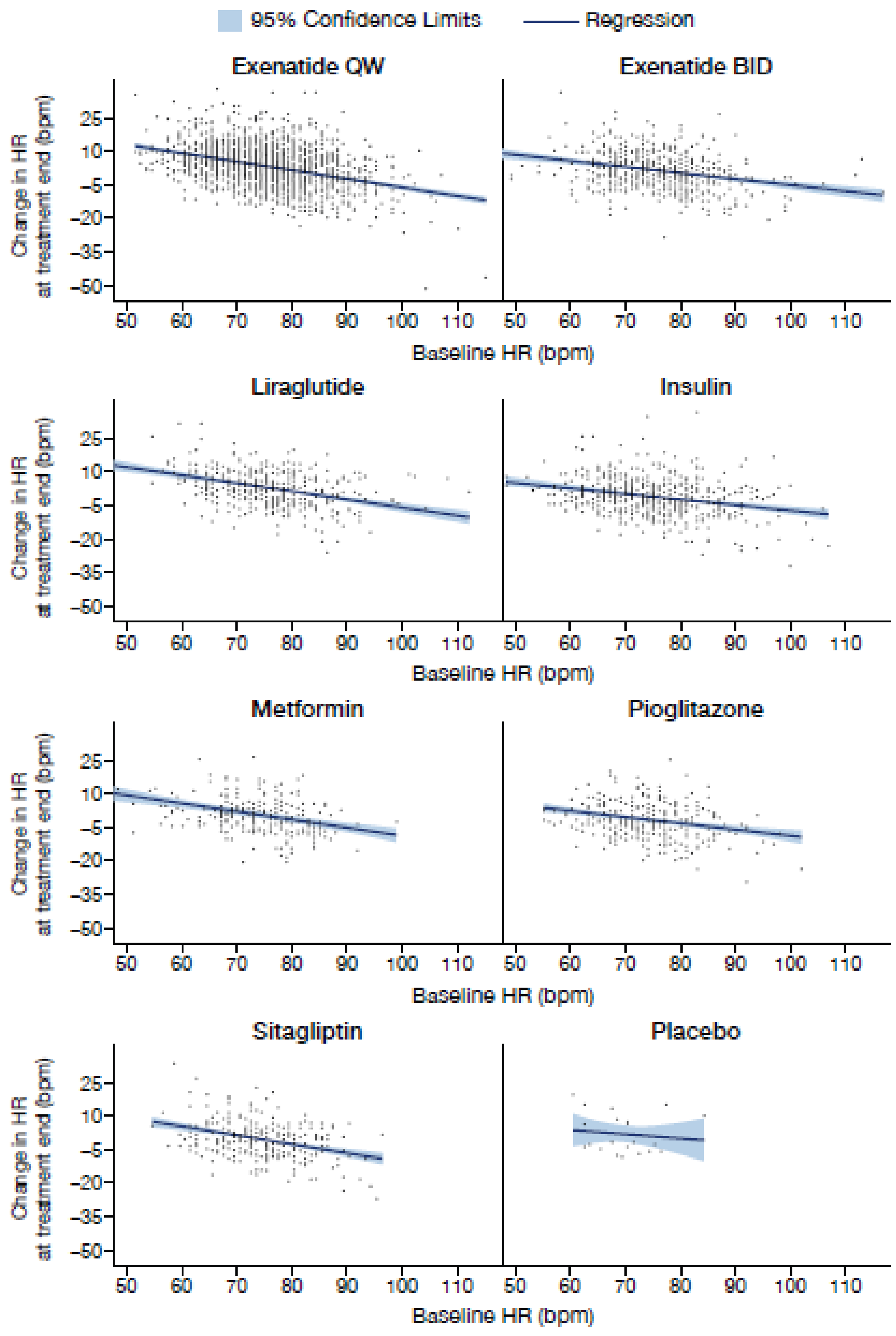

Supplement: Supplementary file 1 — Supplementary material 1 (PDF 352 kb) [file 13300_2018_370_MOESM1_ESM.pdf]
